# 2-year outcomes of phased radiofrequency ablation for atrial fibrillation with the second-generation PVAC Gold ablation catheter

**DOI:** 10.1007/s10840-022-01249-2

**Published:** 2022-05-23

**Authors:** M. N. Klaver, L. I. S. Wintgens, M. C. E. F. Wijffels, V. F. van Dijk, A. Alipour, S. M. Chaldoupi, R. Derksen, J. Peper, J. C. Balt, L. V. A. Boersma

**Affiliations:** 1 Department of Cardiology, St. Antonius Hospital, Nieuwegein, The Netherlands; 2 Department of Cardiology, Amsterdam University Medical Centres, Amsterdam, The Netherlands; 3 Department of Cardiology, Rivierenland Hospital, Tiel, The Netherlands; 4 Department of Cardiology, Maastricht University Medical Centre, Maastricht, The Netherlands; 5 Department of Cardiology, Rijnstate Hospital, Arnhem, The Netherlands

**Keywords:** PVAC Gold, Phased radiofrequency, Atrial fibrillation, Catheter ablation, Pulmonary vein isolation, Long-term performance

## Abstract

**Purpose:**

The second-generation multi-electrode catheter, PVAC Gold, was designed to improve the safe delivery of phased radiofrequency energy using a “single shot” approach for pulmonary vein isolation (PVI), while retaining efficacy. This large registry presents long-term performance in a daily practice setting.

**Methods:**

A total of 1011 patients undergoing first time ablation for atrial fibrillation (AF) using PVAC Gold were included, 639 patients with PVI for paroxysmal AF (PAF PVI) and 372 patients with persistent or long-standing persistent AF, divided into 175 patients receiving PVI only (PersAF PVI) and 197 patients receiving PVI with additional substrate ablation (PersAF PVI +).

**Results:**

At 24-month follow-up, single procedure freedom from atrial tachyarrhythmia (ATA) was 58% (368/639) in the PAF PVI group, 44% (77/175) in the PersAF PVI group, and 29% (57/197) in the PersAF PVI + group. Allowing one repeat procedure in 33% of patients, 76%, 65%, and 54% were free from ATA at 24 months, respectively. Pulmonary vein reconnection was observed in 98% of patients with recurrent arrhythmia after PVI.

**Conclusions:**

Although phased RF ablation with PVAC Gold is quick and safe, the efficacy outcomes are modest compared to current mainstream ablation strategies.

## Introduction

Catheter ablation (CA) of atrial fibrillation (AF) is a well-established treatment option for patients with symptomatic, drug-refractory AF [1, 2]. Conventional point-by-point radiofrequency ablation (RFA) procedures predominantly aim for pulmonary vein isolation (PVI), require high operator skills, and are often lengthy. The multi-electrode phased radiofrequency pulmonary vein ablation catheter (PVAC) was designed to allow circumferential ablation of the pulmonary veins (PV), using an over-the-wire “single shot” approach, and to reduce procedure times, while retaining efficacy and safety. Although successful in achieving these targets, concerns about asymptomatic cerebral embolism (ACE) led to the design of the second-generation multi-electrode catheter, PVAC Gold, to mitigate emboli and improve the delivery of phased RF energy [3]. Compared to the first generation PVAC, PVAC Gold further reduced fluoroscopy, ablation, and total procedure time, while maintaining procedural success [3–5]. Limited data is available evaluating the longer-term efficacy of the second-generation PVAC Gold. This large single-center registry aims to fulfill this knowledge gap and present data on PVAC Gold in an all-comers daily practice setting.

## Methods

### Study design en population

**Fig. 1:**
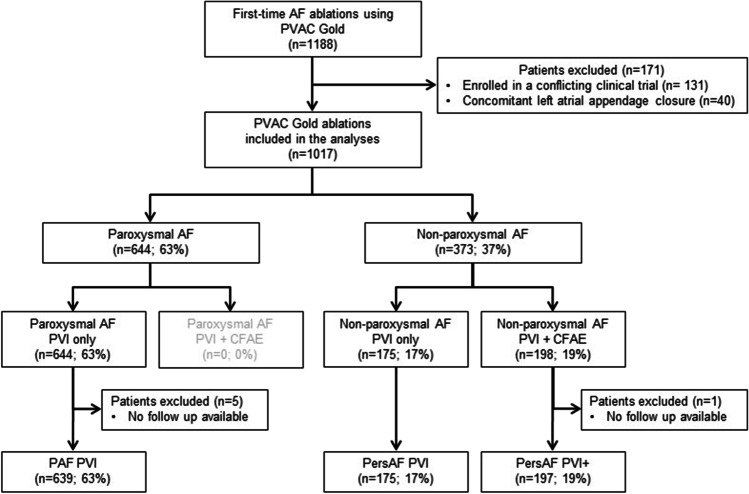
Flowchart: patient inclusion and allocation

The PVAC Gold registry is a retrospective, single-center registry of all consecutive patients undergoing a first-time ablation procedure for AF using the second-generation PVAC Gold phased-RF ablation catheter as strategy of preference. Consecutive patients with paroxysmal AF (PAF), persistent AF (PersAF), and long-standing persistent AF (LS-PersAF) treated between May 2013 (introduction) and December 2016 with the PVAC Gold phased RF ablation system, were enrolled in the study. There were no specific exclusion criteria, and this cohort presents an everyday clinical practice population. Patients enrolled in a conflicting clinical trial or who underwent a concomitant left atrial appendage closure during the same procedure were excluded from analyses. All patients had documented symptomatic AF and were classified according to the 2020 ESC AF guidelines as PAF, PersAF, or LS-PersAF [1, 2]. Patients were allocated to one of three groups. Patients with PAF underwent PVI only (PAF PVI). While patients with PersAF or LS-PersAF were divided into two groups, those who underwent ablation of the PVs only (PersAF PVI) and those who underwent ablation of the PVs with additional ablation of complex fractionated atrial electrograms (CFAE) in the left atrium (PersAF PVI +) (Fig. 1). The decision to perform additional ablation of CFAEs was according to the operator’s discretion.

### Ablation procedure

**Fig. 2:**
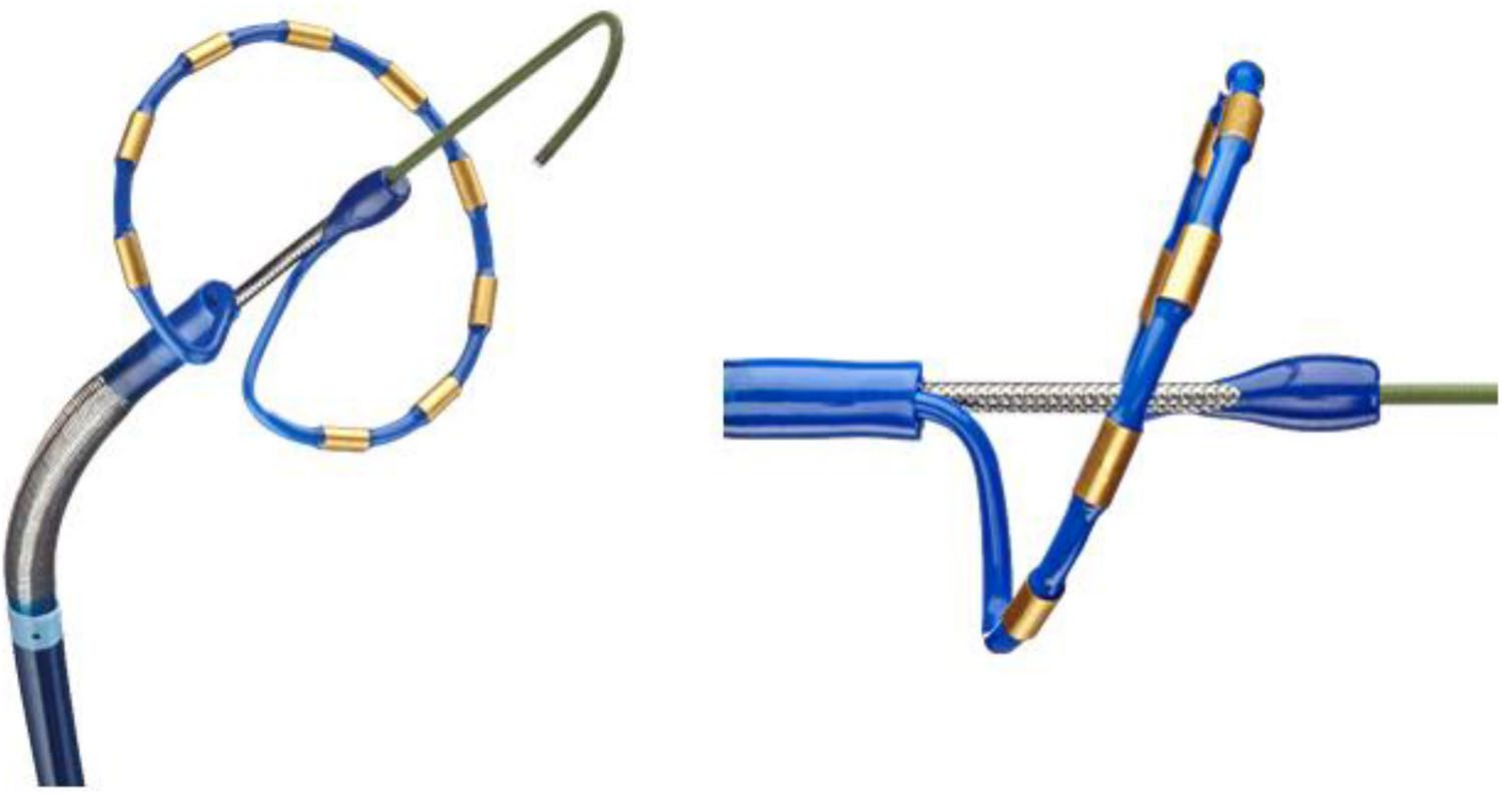
Pulmonary vein ablation catheter (PVAC Gold). PVAC Gold is an over-the-wire, circular, nonpolar mapping, and RF ablation catheter with a 25-mm diameter array at the distal tip, tilted forward 20° to improve tissue contact along the array, and equipped with 9 golden electrodes

The ablation procedure was performed following a standardized ablation protocol as previously described [3, 4, 6]. The PVAC Gold catheter is illustrated in Fig. 2. The catheters are connected to a multichannel, duty cycled RF generator capable of delivering independently powered unipolar energy (to an external pad electrode) and bipolar energy (between adjacent paired electrodes). Energy delivery is titrated up to a target temperature of 60 °C with a maximum output of 8–10 W for 1 min per application. The procedures were performed under continued oral anticoagulation with vitamin K antagonists (target international normalized ratio (INR) between 2.0 and 3.0) or uninterrupted novel oral anticoagulants. Intravenous heparin was used during the procedures to maintain a target activated clotting time ≥ 300 s. Ablation procedures were performed under conscious sedation with intravenous diazepam and fentanyl or, in selected cases, general anesthesia. Ablation of CFAEs was performed using PVAC Gold or the multi-array septal catheter (MASC) and multi-array ablation catheter (MAAC).

### Follow-up

Arrhythmia monitoring was not standardized and left at the discretion of the treating physician. Generally, outpatient visits were scheduled 3, 6, 12, and 24 months after the procedure. An ECG was performed at each visit while Holter (24–72 h) or event monitoring was usually performed when symptoms suggested possible recurrence of arrhythmia. Patients were encouraged to report arrhythmia symptoms and make symptom-initiated ECGs to document recurrence of arrhythmia. When patients were referred back to their general practitioner, they were instructed to present at the outpatient clinic or emergency department if arrhythmia symptoms occurred. After 30–36 months, hospital records were investigated for all follow-up performed, including records from referring hospitals.

### Data collection

Data was collected using a Research Electronic Data Capture (RedCap) system. Custom-built electronic case report forms were designed to capture the data for this registry, incorporating real-time data validation and integrity checks, and to facilitate audits to assure data quality. Information on patient demographics, medical history, procedural characteristics, and follow-up on arrhythmia recurrence, performed diagnostics, repeat ablation procedures (catheter and surgical), and findings at repeat ablation were documented.

### Study endpoints

The primary study outcome was freedom from atrial tachyarrhythmia (ATA) at 24 months after a single procedure using PVAC Gold. Secondary outcomes included freedom from ATA at 24 months after one or two procedures, freedom from ATA at 12 months with or without antiarrhythmic drugs (AAD), and freedom from ATA at 12 months without AAD. The same endpoints were specified for recurrent AF only. For all endpoints, a 90-day blanking period immediately post ablation was incorporated, during which arrhythmia recurrences are not counted toward the endpoint, with the exception of a repeat PVI ablation procedure. Typical right sided flutter and atrioventricular node re-entry tachycardia were not considered an endpoint. Recurrence of ATA and AF was defined as any documented episode of ATA or AF by any form of monitoring, regardless of symptoms. Patients that became asymptomatic with no documented AF during FU were occasionally discharged from active surveillance. These patients were considered free from recurrent ATA at 24 months when hospital records showed no documentation of recurrent ATA and no patient-initiated visits had been performed until the time of review (30–36 months). In patients undergoing a repeat electrophysiology study, the number and distribution of reconnection of the PVs was assessed.

### Statistical analysis

Data is presented as mean and standard deviation for continuous variables, as median and interquartile range for not normally distributed continuous variables, and as numbers and percentages for categorical variables. Continuous data were compared using parametric or nonparametric tests when appropriate. Categorical data were compared using *χ*^2^ test or Fisher’s exact test when a small number of events were observed. The probability of ATA and AF-free survival was estimated by Kaplan–Meier analyses. The log rank test was used to determine any significant differences between groups and risk stratification were evaluated using univariate and multivariate Cox proportional hazard analyses. Data were included when the *p*-value was < 0.10. A backward stepwise approach was used. All tests were two-tailed and the limit for statistical significance was set at *p* < 0.05. Statistical analyses were performed using R software, version 3.6.3 (R Foundation for Statistical Computing) and SPSS for Windows software, version 26 (SPSS Inc., Chicago, IL, USA).

## Results

A total of 1017 patients were treated using PVAC Gold and follow-up documentation was available for almost all (1011, 99.4%) patients. Enrolment, inclusion for analyses, and treatment group allocation are illustrated in Fig. 1. The baseline characteristics are shown in Table 1. Patients in the PAF PVI group were more often female, had a lower BMI, a more preserved left atrial volume, and less heart failure or prior stroke compared to the non-paroxysmal AF patients (all *p* < 0.05). In the non-paroxysmal AF groups, the PersAF PVI + group was older (*p* = 0.040), had more often LS-PersAF (*p* = 0.002), and more mitral valve regurgitation (*p* = 0.003) compared to the PersAF PVI group. Ninety-eight percent of all patients had failed one or more class I or III AAD prior to ablation and no patients had undergone previous CA or surgical ablation (SA) for AF in any of the groups. The experience with PVAC Gold procedures per operator was similar in all cohorts.

**Table 1:** Baseline characteristics

Demographics	PAF PVI *n* = 639	PersAF PVI *n* = 175	PersAF PVI + *n* = 197	Overall *p*-value
Gender male	66.6%	78.9%	78.3%	< 0.001
Age (years), mean ± SD	61.1 ± 10.5	61.0 ± 10.6	63.1 ± 9.4	0.045
BMI (kg/m^2^), mean ± SD	26.6 ± 4.0	27.4 ± 3.6	27.9 ± 4.5	< 0.001
Type of AF				
Paroxysmal	100%	-	-	
Persistent	-	95.4%	86.3%	0.002
Long-standing persistent	-	4.6%	13.7%	0.002
CHA_2_DS_2_-VASC, mean ± SD	1.6 ± 1.3	1.8 ± 1.5	1.9 ± 1.6	0.008
HASBLED, median [IQR]	1 [0–2]	1 [0–2]	1 [0–2]	0.003
Left atrial volume index				
Normal < 35 ml/m^2^	67.9%	46.6%	39.3%	< 0.001
Mildly dilated 35–41 ml/m^2^	20.8%	34.2%	38.7%	< 0.001
Moderately dilated 42–48 ml/m^2^	6.3%	9.3%	9.9%	< 0.001
Severely dilated > 48 ml/m^2^	5.6%	10.6%	12.0%	< 0.001
Reduced LVEF < 50%	8.3%	21.9%	28.0%	< 0.001
Mitral valve regurgitation grade ≥ 2	7.2%	9.1%	16.4%	0.001
Prior AAD failed	98.0%	100%	97.3%	0.127
History of atrial flutter	20.0%	22.9%	15.7%	0.212
Prior CTI ablation	6.4%	5.1%	3.6%	0.299
	PAF PVI	PersAF PVI	PersAF PVI +	*p*-value
Comorbidities				
Congestive heart failure	4.3%	14.3%	13.6%	< 0.001
Hypertension	39.8%	46.3%	50.0%	0.024
Diabetes	5.9%	6.9%	9.1%	0.289
Stroke/TIA	4.7%	10.9%	11.6%	< 0.001
Vascular disease	16.3%	20.6%	14.6%	0.279
OSAS	4.8%	4.0%	7.6%	0.227
Reduced kidney function (GFR < 60)	7.9%	9.1%	12.3%	0.194
Procedure				
Standard 4 PVs anatomy	83.4%	85.7%	81.3%	0.522
LCPV	12.6%	12.0%	13.6%	0.886
RMPV	5.3%	2.3%	6.6%	0.147
Acute success (all PVs isolated)	97.7%	95.4%	99.0%	0.084

*BMI*, body mass index; *SD*, standard deviation; *IQR*, interquartile range; *AF*, atrial fibrillation; *LVEF*, left ventricular ejection fraction; *TIA*, transient ischemic attack; *OSAS*, obstructive sleep apnea syndrome; *GFR*, glomerular filtration rate; *PV*, pulmonary vein; *LCPV*, left common pulmonary vein; *RMPV*, right middle pulmonary vein.

*p*-values are for the overall comparisons among the three groups. Post hoc pairwise multiple comparisons are provided in the supplementary materials, supplementary table 1.

### Atrial tachyarrhythmia-free survival after a single procedure

**Fig. 3:**
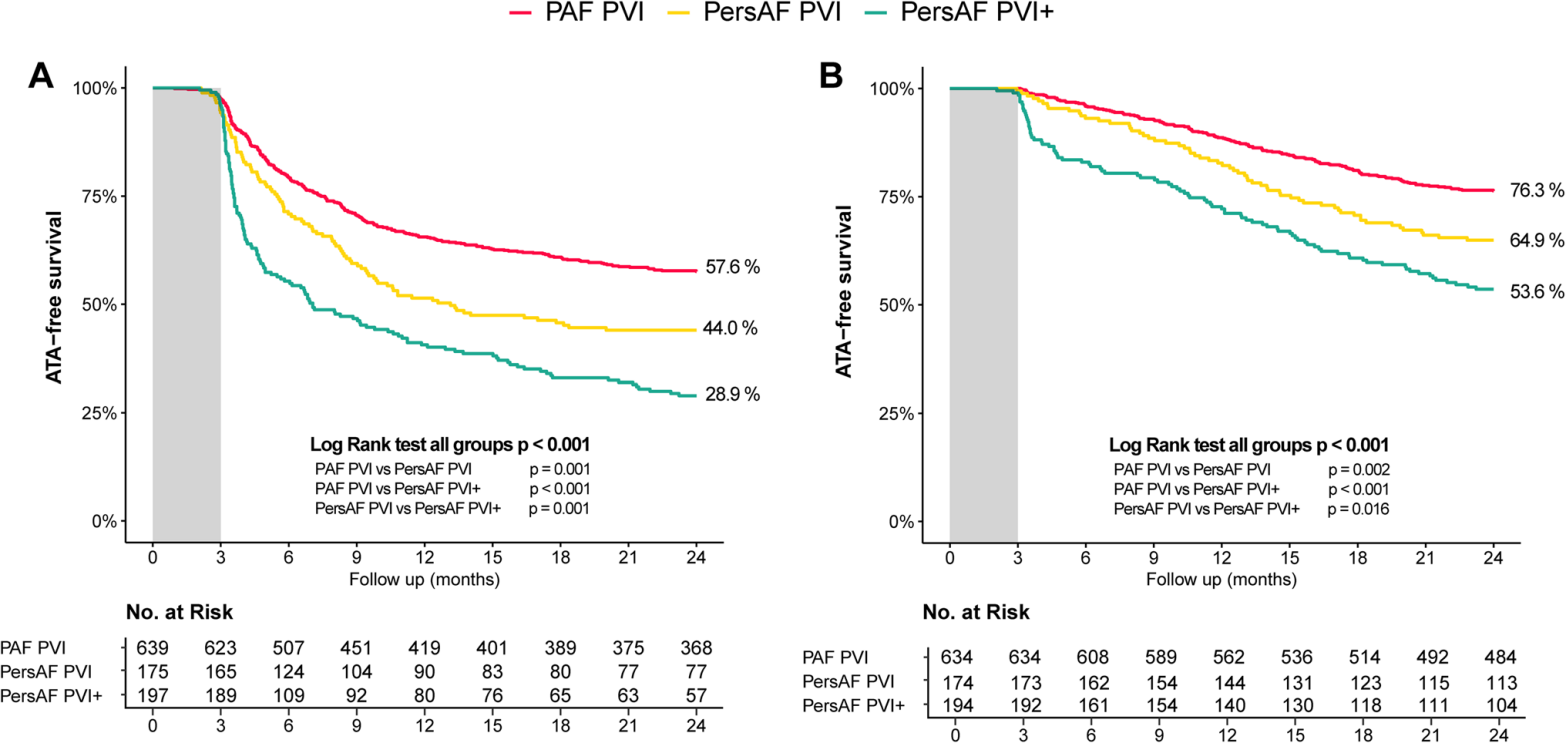
Atrial tachyarrhythmia-free survival. ATA-free survival after (**A**) a single procedure and (**B**) after a mean of 1.3 procedures

Arrhythmia outcomes are presented in Fig. 3 and Table 2. At 24 months, ATA-free survival after a single procedure allowing the use of AAD was observed in 368 of 639 patients (57.6%) in the PAF PVI group, 77 of 175 patients (44.0%) in the PersAF PVI group, and 57 of 197 patients (28.9%) in the PersAF PVI + group. PAF PVI patients showed significantly better outcomes than the non-PAF groups, while PersAF PVI had significantly better outcomes than PersAF PVI + patients (*p* < 0.001 for the overall comparison among the groups. Post hoc pairwise comparisons between groups were all significantly different (*p* ≤ 0.001). Adding CFAE ablation to the PVI did not seem to improve outcome, presumptively reflecting the higher co-morbidity disease state of the latter cohort. Recurrence of ATA was observed after a median of 5.6 months [3.6–9.5], comparable between all groups (*p* = 0.137). In the PAF PVI group, the majority (82%) remained paroxysmal while 18% progressed to persistent AF. In the PersAF PVI group, 66% of recurrences changed to paroxysmal AF, while in the PersAF PVI + group, 30% of recurrences AF changed to paroxysmal AF. One or more Holter tests were performed in 49% of patients. Adjusted for baseline characteristics, ATA-free survival remained different between groups in favor of the PAF PVI group compared to the PersAF PVI group (HR 1.550, 95%CI 1.228–1.957, *p* < 0.001) and compared to the PersAF PVI + group (HR 2.385, 95%CI 1.938–2.935, *p* < 0.001), as well for PersAF PVI compared to PersAF PVI + (HR 1.538, 95%CI 1.188–1.992, *p* = 0.001). Independent predictors for ATA recurrence from the Cox proportional hazard model were age (HR 1.014 per year, 95%CI 1.004–1.023, *p* = 0.001) and female gender (HR 1.259, 95%CI 1.039–1.526, *p* = 0.019).

**Table 2:** Arrhythmia endpoints

Endpoint	Follow-up (months)	PVI	AAD	PAF PVI (*n* = 639)	PersAF PVI (*n* = 175)	PersAF PVI + (*n* = 197)	Overall *p*-value
Freedom from ATA	12	**1**	with or without	419 (65.6%)	90 (51.4%)	80 (40.6%)	< 0.001
Freedom from AF	12	**1**	with or without	427 (66.8%)	95 (54.3%)	81 (41.1%)	< 0.001
Freedom from ATA	12	**1**	without	317 (50.7%)	72 (41.9%)	62 (32.5%)	< 0.001*
Freedom from AF	12	**1**	without	319 (51.0%)	73 (42.4%)	63 (33.0%)	< 0.001*
Freedom from ATA	24	**1**		368 (57.6%)	77 (44.0%)	57 (28.9%)	< 0.001
Freedom from AF	24	**1**		376 (58.8%)	80 (45.7%)	57 (28.9%)	< 0.001
Freedom from ATA	24	**1 or 2**		484 (76.3%)	113 (64.9%)	104 (53.6%)	< 0.001
Freedom from AF	24	**1 or 2**		494 (77.9%)	117 (67.2%)	106 (54.6%)	< 0.001

*PVI*, pulmonary vein isolation; *AAD*, anti-arrhythmic drugs; *AF*, atrial fibrillation; *ATA*, atrial tachyarrhythmia.

*p*-values are for the overall comparisons among the three groups. Post hoc pairwise multiple comparison showed all between group comparisons were significant (*p* < 0.05), with the exception between PersAF PVI and PersAF PVI + (marked with asterisk) for freedom from ATA without the use of AAD and freedom from AF at 12 months without the use of AAD (*p* = 0.063 and 0.064, respectively). Provided in supplementary table 2.

### Other efficacy outcomes

**Fig. 4:**
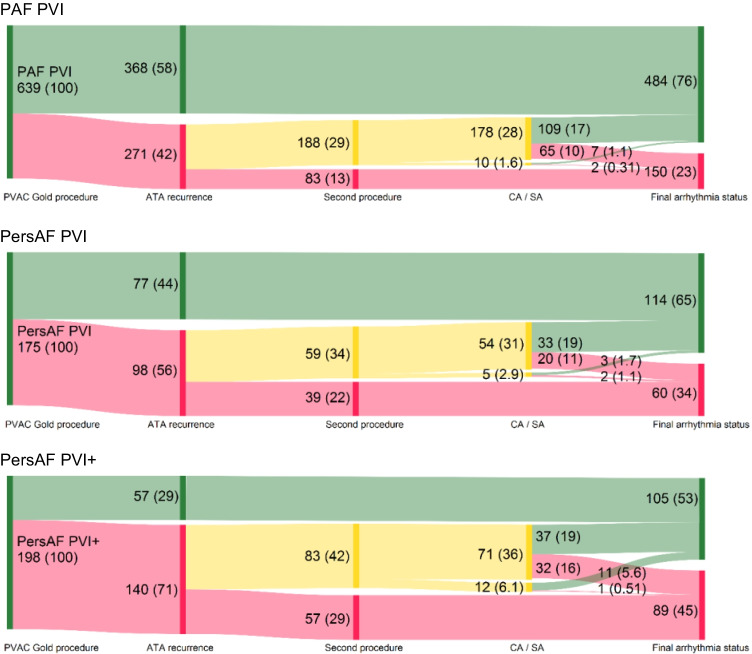
Directional flow diagram presenting arrhythmia status after PVAC Gold PVI, allowing 1 repeat procedure. Number and percentage (in parentheses) of patients are presented. Node and path size represent the percentage of patients. Node and path color represent arrhythmia status, with ATA-free survival in green and ATA recurrence in red. Yellow nodes and paths represent patients with initial recurrence who underwent a second ablation procedure. ATA, atrial tachyarrhythmia; CA, catheter ablation; SA, surgical ablation

Figure 3B and Table 2 additionally present the secondary efficacy outcomes at 12- and 24-month follow-up for ATA-free survival and AF-free survival after one or two procedures, on AADs, and off AADs. For all efficacy outcomes, consistent results were seen, showing the most favorable outcomes in the PAF PVI group, followed by PersAF PVI group and the lowest in the PersAF PVI + group (*p* < 0.05 for all comparisons between groups, except for the comparison between PersAF PVI and PersAF PVI + for ATA-free survival and AF-free survival at 12 months off AAD (*p* = 0.064 and 0.063, respectively) (supplementary table 2). While free from recurrent ATA, 22.0% were still on antiarrhythmic medication at 12-month follow-up (22.9%, 19.1%, and 20.5% respectively, *p* = 0.698). Figure 4 presents a visualization of patients in each group, their ablation treatment, and arrhythmia status after up to 24 months of follow-up (directional flow diagram).

A second procedure was performed in 330 patients, 32.6% of the total population and 64.8% of patients with recurrent arrhythmia. CA was performed in 303 patients (91.8%) and SA in 27 patients (8.2%). For the entire cohort, repeat ablation was performed significantly more often in the PersAF PVI + group compared to the PersAF PVI and PAF PVI groups (42.1% versus 33.7% and 29.4%, respectively (*p* = 0.004). However, in patients with ATA recurrence, repeat ablation was performed equally in all groups (59.3% vs 60.2%, and 69.4% respectively, *p* = 0.072). Repeat CA or SA was performed after a median 9 [6–13], 10 [6–13], and 11 [7–15] months for the PAF PVI, PersAF PVI, and PersAF PVI + groups, respectively (*p* = 0.009). Repeat catheter ablation was performed predominantly using point-by-point RFA (99.0%). In patients with initial recurrence, after a single repeat ablation procedure, 63.4% in the PAF PVI, 62.1% in the PersAF PVI, and 58.8% in the PersAF PVI + group were free from recurrent ATA afterwards (*p* = 0.776). At 24-month follow-up, after a mean of 1.3 procedures, 76.3% in the PAF PVI, 64.9% in the PersAF PVI, and 53.6% in the PersAF PVI + were free from ATA recurrence (overall *p* < 0.001, between groups all *p* < 0.05) (Fig. 3B).

**Fig. 5:**
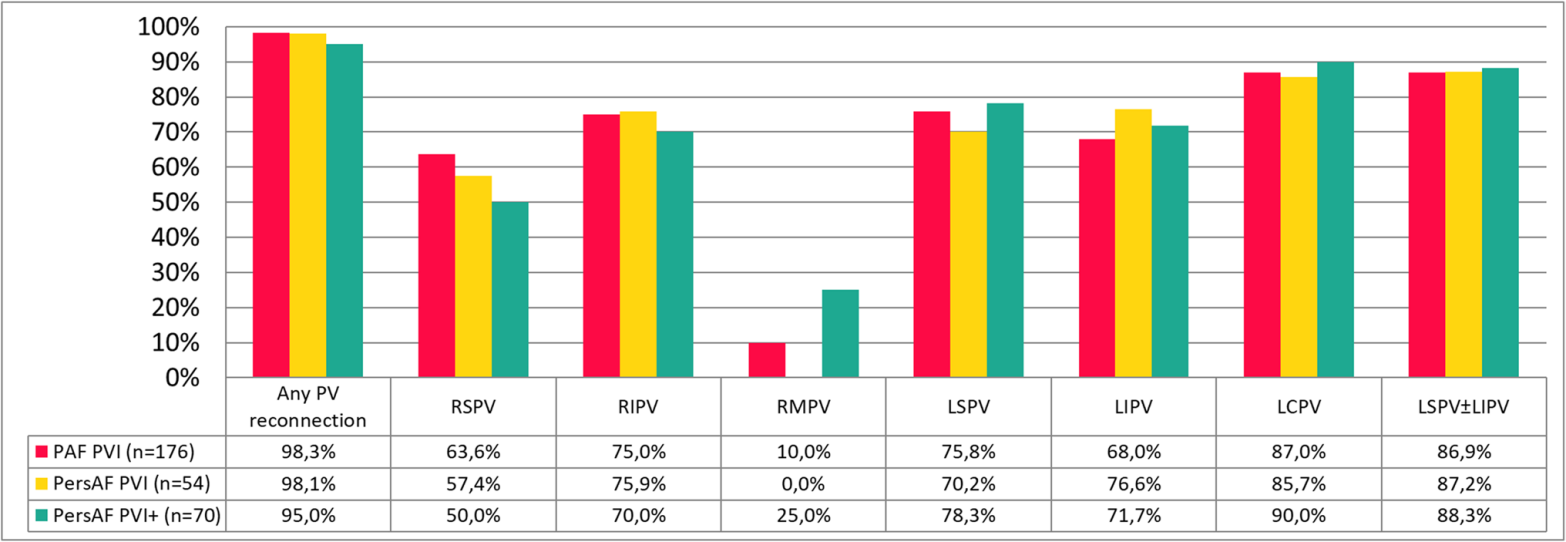
Reconnection of pulmonary veins during repeat electrophysiology study after a single PVAC Gold procedure. Histogram presenting the percentage of reconnection found for each pulmonary vein. PV, pulmonary veins; RSPV, right superior pulmonary vein; RIPV, right inferior pulmonary vein; RMPV, right middle pulmonary vein; LSPV, left superior pulmonary vein; LIPV, left inferior pulmonary vein; LCPV, left common pulmonary vein; LSPV ± LIPV, reconnection in either or both the LSPV and LIPV

Figure 5 shows the findings during repeat electrophysiology study, available in 300 out of 303 repeat catheter ablation procedures. Electrical reconnection of one or more of the PVs (average 2.7 ± 1.1) was seen in 295 (98%) patients, equally distributed between groups (*p* = 0.517). The number of patients with no, 1, 2, 3, and 4 reconnected PVs was 5 (2%), 34 (11%), 88 (29%), 86 (29%), and 87 (29%), respectively, and comparable between groups (*p* = 0.886). Reconnection of the LSPV, LIPV, LCPV, RSPV, RIPV, and RMPV was seen in 196 (75%), 183 (70%), 35 (88%), 178 (59%), 222 (74%), and 2 (13%) respectively, comparable between groups (*p* > 0.05 for all comparisons). The LCPV showed a higher rate of reconnection compared to individual LSPV or LIPV at redo procedure, but not when compared to LSPV and/or LIPV combined (87.3% vs 87.5%; *p* = 0.973).

## Discussion

In our registry, we present data in a large all comers’ population with PAF, PersAF, and LS-PersAF. Following our previous paper demonstrating the favorable procedural and safety outcomes [4], we present performance outcomes up to 24 months, on and off AAD, as well as findings during repeat procedures in symptomatic patients.

Compared to prior observational studies reporting on PVAC Gold, the efficacy outcomes in our study were more modest. The multicenter GOLD AF study and a German cohort study reported 12-month success rates of 71–82% for PAF and 62–68% for PersAF [7, 8]. These studies similarly did not include a standardized or scrutinous arrhythmia assessment during follow-up. Compared to the FIRE&ICE trial, at 12 months, PVI using PVAC Gold in our PAF cohort was less effective (50.7%) than point-by-point RF PVI (64%) and cryoballoon PVI (65%) in the FIRE&ICE study [9]. To some extent, the lower freedom of arrhythmias may be due to the non-selected, all-comers population, including patients with less favorable demographics that are usually excluded in randomized controlled clinical trials (e.g., elderly, heart failure, large left atrium, valvular disease, or obese). Our large single-center cohort is the first to report on longer-term follow-up beyond 12 months using PVAC Gold, showing a further decline in freedom of ATA at 24 months to 58% in PAF and 44% in PersAF receiving only PVI and 29% in PersAF patients receiving PVI with additional lesions.

Prior randomized head-to-head studies comparing PVAC phased RFA to point-by-point single-tip RFA or cryoballoon ablation (CBA) are limited and relatively small, and have focused only on PAF patients. The MYSTIC PAF study, randomizing patients to either first generation PVAC or point-by-point irrigated RF PVI, failed to meet non-inferiority at 12 months but showed numerically very similar success rates (76% for PVAC vs 81% for RFA) [10]. The multicenter Gold Force trial randomized 208 patients toward PVAC Gold or RFA with contact force sensing and demonstrated significantly more AF recurrence in the PVAC Gold group (46.6% versus 26.2%) [11]. A smaller single-center randomized study in 42 patients compared PVAC Gold with the second-generation cryoballoon and was prematurely terminated at interim analysis because of superiority of PVI using cryoballoon (86% vs 50%, *p* < 0.05) [12]. Although these studies all showed the single-shot PVAC approach to have more favorable and significantly shorter procedure times, the efficacy outcomes are all less favorable.

Short procedure times have been a consistent benefit of PVAC ablation in comparison to other ablation systems. A network meta-analysis of historical data between 2010 and 2017 on single tip RFA, CBA, laser energy, and PVAC showed procedure time and fluoroscopy time using PVAC to be superior to all other catheters, while efficacy and safety were similar [13]. However, since the introduction of contact force sensing and ablation index guided PVI with a strict protocol for ablation application density by using the CLOSE protocol, success rates have further improved for point-by-point ablation. One year freedom from ATA in PAF patients receiving PVI was reported to be as high as 87–94% and 78% at 2 years, while ATA burden decreased by a median 100%, and procedure times were shorter compared to conventional RFA [14, 15]. Similarly, the second-generation cryoballoon in the STOP AF study showed improved outcomes with a 1-year efficacy of 82%, 74% at 2 years, and 68% at 3 years [16]. More recent meta-analysis investigating these improved ablation strategies show further reduced procedural times, while improving performance compared to first generation RFA and CBA [17]. One could argue that there may be some merit to a trade-off between significantly shorter procedures versus improved performance, when outcome differences remain relatively small like in the MYSTIC PAF trial. Also, it may not be necessary to achieve complete freedom of ATA as long as similar symptom or burden reduction is achieved compared to other ablation strategies. However, it seems reasonable to assume that a lower complete freedom of ATA may lead to more morbidity, hospital visits, and repeat procedures. Recently, pulsed field ablation (PFA) has been introduced as a promising new modality for ablation, with 87% of PAF patients free from arrhythmias at 12 months in a first pilot study with drastically shorter procedure times [18]. It seems that the rise of all these improved ablation strategies leaves little to no future prospects for PVAC phased RF ablation as a future tool for PVI.

The main reason for this appears to be the long-term effectiveness of PVI. Durable PVI remains the cornerstone of AF ablation and recurrence of AF is attributed to electrical reconnection of the PVs. In patients with recurrent AF, during repeat PVI procedure, we found 98% of patients showed reconnection in one or more PVs, mean 2.7 ± 1.1, comparable to reports from the first generation PVAC [8]. Comparison between RFA and CBA was done in the FIRE&ICE redo study, concluding that patients with one or more reconnected vein(s) were distributed equally (83% for RFA and 78% for CBA), although the number of reconnected veins was higher in the RFA cohorts [19]. Wieczorek et al. investigated reconnection after PVAC and 2nd generation CBA, and although comparable high rates of reconnection in patients with recurrent AF were seen, significantly more PVs showed reconnection in the PVAC group [20]. De Pooter et al. showed that in patients after CLOSE-guided PVI, during repeat ablation in only 48% of patients one or more PVs showed reconnection [14]. Similar results were seen after the 2nd generation CBA, where only in 55% of patients one or more PV showed reconnection [21]. Finally, early results from PFA show 100% durability of PVI in the most recent patient cohorts [18]. In our cohort, PV reconnection rates were usually higher during redo procedures while also the number of reconnected PVs appears to be high compared to other ablation strategies, suggesting inferior durable PV isolation using PVAC Gold phased RF ablation. Possible explanations could be the more ostial ablation position using PVAC Gold compared to the more antral ablation using cryoballoon or RFA. Furthermore, no contact force sensing is available on the PVAC Gold platform, limiting the feedback for the operator on lesion formation or the ability to use lesion size index targets. Finally, animal studies showed phased RF using PVAC created lesions with limited width (2.2 mm) [22] and modifications to the phased RF generator to prevent power and temperature overshooting, may have hampered the creation of transmural and durable lesions, yet there are no studies investigating this.

In line with STAR AF II, we found a higher percentage of patients with recurrent arrhythmia after additional CFAE ablations compared to PVI only [23]. The PersAF cohort that received substrate ablation in addition to PVI was different at baseline with more co-morbidity, higher AF burden or in chronic AF, and had more enlarged left atria. Although additional CFAE ablation is safe [4], it does not appear to provide benefit over PVI alone for arrhythmia-free survival.

### Future perspectives

The PVAC catheter using phased RF has been extensively used and was widely adopted. When it was associated with a higher rate of asymptomatic cerebral embolism due to interaction of the proximal and distal electrode on the circular array, the second-generation PVAC Gold was modified to optimize tissue contact for optimal energy delivery, and avoid interaction between the proximal and distal electrodes of the array by taking away electrode 10, while the generator algorithm was changed to more conservative settings preventing power and temperature overshooting [3]. Previous studies demonstrated that these modifications reduced ACE rates, ablation times, and procedure times, while retaining acute success and safety [3, 7]. Nevertheless, the modifications to the catheter and the more conservative phased RF generator algorithm may have hampered the durability of PV isolation and arrhythmia-free survival, as seen in this study. FDA approval for phased RF ablation was never obtained, and there has been no development to further improve phased radiofrequency ablation platform. Meanwhile, PFA is rapidly evolving as a potentially faster and safer alternative to thermal ablation strategies. The PVAC Gold ablation system was modified to be able to deliver biphasic, bipolar pulsed field ablation simultaneously to all electrodes for a single shot approach or user-selected electrodes. The Pulsed AF study (NCT04198701) is a prospective, multi-center, non-randomized, worldwide pre-market clinical study that will provide insights into the Medtronic pulsed field ablation system for PVI using the PVAC Gold catheter [24]. First-in-human pilot trial results showed a promising 100% acute PVI rate with no serious adverse events attributed to the PFA technology within the first 30 days post procedure [24].

### Limitations

The main limitations of the study arise from the non-randomized, single-center, retrospective design. While providing daily practice outcomes, absence of a scrutinous rhythm follow-up hampers the detection of asymptomatic or less frequent ATA recurrence.

## Conclusion

Although phased RF ablation with PVAC Gold is quick and safe, the efficacy outcomes during 2-year follow-up are modest. Compared to ablation index guided RFA and latest generation CBA, the use of PVAC Gold as an equal first-choice strategy for PVI may no longer be justified.

## Supplementary Information

ESM 1 (DOCX 20.3 KB)
