# Supplementary material for: 2-year outcomes of phased radiofrequency ablation for atrial fibrillation with the second-generation PVAC Gold ablation catheter
Source: J Interv Card Electrophysiol. 2022 May 23;65(2):471–80. doi: 10.1007/s10840-022-01249-2 (PMC9640466; doi:10.1007/s10840-022-01249-2)
Supplement: Supplementary file 1 — (DOCX 20.3 KB) [file 10840_2022_1249_MOESM1_ESM.docx]

**Supplementary data**

**Supplementary table 1.** Baseline characteristics including pairwise comparisons

| **Demographics** | **PAF PVI (A) n = 639** | **PersAF PVI (B) n = 175** | **PersAF PVI+ (C)**  **n = 197** | **p-value** | p-value  A vs B | p-value  A vs C | | p-value  B vs C |
| --- | --- | --- | --- | --- | --- | --- | --- | --- |
| Gender male | 66.6% | 78.9% | 78.3% | <0.001 | 0.002 | 0.002 | 0.873 | |
| Age (years), mean ± SD | 61.1±10.5 | 61.0±10.6 | 63.1±9.4 | 0.045 | 0.889 | 0.016 | 0.040 | |
| BMI (kg/m^2^), mean ± SD | 26.6±4.0 | 27.4±3.6 | 27.9±4.5 | <0.001 | 0.015 | <0.001 | 0.291 | |
| Type of AF |  |  |  |  |  |  |  | |
| Paroxysmal | 100% | - | - |  |  |  |  | |
| Persistent | - | 95.4% | 86.3% | 0.002 |  |  |  | |
| Long-standing persistent | - | 4.6% | 13.7% | 0.002 |  |  |  | |
| CHA_2_DS_2_-VASC, mean ± SD | 1.6±1.3 | 1.8±1.5 | 1.9±1.6 | 0.008 | 0.122 | 0.040 | 0.715 | |
| HASBLED, median [IQR] | 1 [0 – 2] | 1 [0 – 2] | 1 [0 – 2] | 0.003 | 0.058 | 0.006 | 0.621 | |
| LAVI |  |  |  |  |  |  |  | |
| Normal <35 ml/m^2^ | 67.9% | 46.6% | 39.3% | <0.001 | <0.001 | <0.001 | 0.652 | |
| Mildly dilatated 35-41 ml/m^2^ | 20.8% | 34.2% | 38.7% | <0.001 |  |  |  | |
| Moderately dilatated 42-48 ml/m^2^ | 6.3% | 9.3% | 9.9% | <0.001 |  |  |  | |
| Severely dilatated >48 ml/m^2^ | 5.6% | 10.6% | 12.0% | <0.001 |  |  |  | |
| Reduced LVEF <50% | 8.3% | 21.9% | 28.0% | <0.001 | <0.001 | <0.001 | 0.181 | |
| Mitral valve regurgitation grade ≥2 | 7.2% | 9.1% | 16.4% | 0.001 | 0.409 | <0.001 | 0.043 | |
| Prior AAD failed | 98.0% | 100% | 97.3% | 0.127 |  |  |  | |
| History of atrial flutter | 20.0% | 22.9% | 15.7% | 0.212 |  |  |  | |
| Prior CTI ablation | 6.4% | 5.1% | 3.6% | 0.299 |  |  |  | |
| **Comorbidities** | **PAF PVI** | **PersAF PVI** | **PersAF PVI+** | **p-value** |  |  |  | |
| Congestive heart failure | 4.3% | 14.3% | 13.6% | <0.001 | <0.001 | <0.001 | 0.872 | |
| Hypertension | 39.8% | 46.3% | 50.0% | 0.024 | 0.111 | 0.012 | 0.505 | |
| Diabetes | 5.9% | 6.9% | 9.1% | 0.289 |  |  |  | |
| Stroke/TIA | 4.7% | 10.9% | 11.6% | <0.001 | 0.002 | <0.001 | 0.803 | |
| Vascular disease | 16.3% | 20.6% | 14.6% | 0.279 |  |  |  | |
| OSAS | 4.8% | 4.0% | 7.6% | 0.227 |  |  |  | |
| Reduced kidney function (GFR <60) | 7.9% | 9.1% | 12.3% | 0.194 |  |  |  | |
| **Procedure** | **PAF PVI** | **PersAF PVI** | **PersAF PVI+** | **p-value** |  |  |  | |
| Standard 4 PVs anatomy | 83.4% | 85.7% | 81.3% | 0.522 |  |  |  | |
| LCPV | 12.6% | 12.0% | 13.6% | 0.886 |  |  |  | |
| RMPV | 5.3% | 2.3% | 6.6% | 0.147 |  |  |  | |
| Acute success (all PVs isolated) | 97.7% | 95.4% | 99.0% | 0.084 |  |  |  | |
| BMI, body mass index; SD, standard deviation; IQR, interquartile range; AF, atrial fibrillation; LVEF, left ventricular ejection fraction; TIA, transient ischemic attack; OSAS, obstructive sleep apnea syndrome; GFR, glomerular filtration rate; PV, pulmonary vein; LCPV, left common pulmonary vein; RMPV, right middle pulmonary vein.  P values are for the overall comparisons among the three groups. Post-hoc, pairwise multiple comparison, are provided in the supplementary materials. | | | | | | | | |

**Supplementary table 2.** Arrhythmia endpoints including pairwise comparisons

| **Endpoint** | **Follow up (months)** | **PVI** | **AAD** | **PAF PVI (A) (n=639)** | **PersAF PVI (B) (n=175)** | **PersAF PVI+ (C)**  **(n=197)** | **p-value** | **p-value**  **A vs B** | **p-value**  **A vs C** | **p-value**  **B vs C** |  |
| --- | --- | --- | --- | --- | --- | --- | --- | --- | --- | --- | --- |
| Freedom from ATA | 12 | **1** | **+/-** | 419 (65.6%) | 90 (51.4%) | 80 (40.6%) | <0.001 | 0.001 | <0.001 | 0.007 |  |
| Freedom from AF | 12 | **1** | **+/-** | 427 (66.8%) | 95 (54.3%) | 81 (41.1%) | <0.001 | 0.002 | <0.001 | 0.002 |  |
| Freedom from ATA | 12 | **1** | **-** | 317 (50.7%) | 72 (41.9%) | 62 (32.5%) | <0.001 | 0.040 | <0.001 | 0.064 |  |
| Freedom from AF | 12 | **1** | **-** | 319 (51.0%) | 73 (42.4%) | 63 (33.0%) | <0.001 | 0.046 | <0.001 | 0.063 |  |
| Freedom from ATA | 24 | **1** |  | 368 (57.6%) | 77 (44.0%) | 57 (28.9%) | <0.001 | 0.001 | <0.001 | 0.001 |  |
| Freedom from AF | 24 | **1** |  | 376 (58.8%) | 80 (45.7%) | 57 (28.9%) | <0.001 | 0.001 | <0.001 | <0.001 |  |
| Freedom from ATA | 24 | **1 or 2** |  | 484 (76.3%) | 113 (64.9%) | 104 (53.6%) | <0.001 | 0.002 | <0.001 | 0.016 |  |
| Freedom from AF | 24 | **1 or 2** |  | 494 (77.9%) | 117 (67.2%) | 106 (54.6%) | <0.001 | 0.002 | <0.001 | 0.008 |  |
| PVI, pulmonary vein isolation; AAD, anti-arrhythmic drugs; AF, atrial fibrillation; ATA, atrial tachyarrhythmia  P values are for the overall comparisons among the three groups. Post hoc, pairwise multiple comparison showed all between group comparisons were significant, with the exception of freedom from ATA without the use of AAD and freedom from AF at 12 months without the use of AAD between PersAF PVI and PersAF PVI+ (p= 0.063 and 0.064, respectively). | | | | | | | | | | | |
